# Low Levels of Adenosine and GDNF Are Potential Risk Factors for Parkinson’s Disease with Sleep Disorders

**DOI:** 10.3390/brainsci13020200

**Published:** 2023-01-24

**Authors:** Li Wang, Zheng Gao, Gang Chen, Deqin Geng, Dianshuai Gao

**Affiliations:** 1 School of Basic Medical Sciences, Nanjing Medical University, Nanjing 211166, China; 2 Department of Neurology, The Affiliated Hospital of Xuzhou Medical University, Xuzhou 221006, China; 3 The First Clinical College, Xuzhou Medical University, Xuzhou 221004, China; 4 Department of Neurology, The Affiliated Shuyang Hospital of Traditional Chinese Medicine of Yangzhou Medical University, Suqian 223600, China; 5 Department of Neurobiology, Xuzhou Key Laboratory of Neurobiology, Xuzhou Medical University, Xuzhou 221004, China

**Keywords:** Parkinson’s disease, sleep disorders, adenosine, GDNF, neurotransmitters

## Abstract

Sleep disturbances are the most prevalent non-motor symptoms in the preclinical stage of Parkinson’s disease (PD). Adenosine, glial-derived neurotrophic factor (GDNF), and associated neurotransmitters are crucial in the control of sleep arousal. This study aimed to detect the serum levels of adenosine, GDNF, and associated neurotransmitters and explored their correlations with PD with sleep disorders. Demographic characteristics and clinical information of PD patients and healthy participants were assessed. Serum concentrations of adenosine, GDNF, and related neurotransmitters were detected by ELISA and LC-MS. The correlation between serum levels of adenosine, GDNF, and associated neurotransmitters and sleep disorders was explored using logistic regression. PD patients with sleep disorders had higher scores of HAMA, HAMD, ESS, UPDRS-III, and H-Y stage. Lower levels of adenosine, GDNF, and γ-GABA were observed in PD patients who had sleep problems. Logistic regression analysis showed adenosine and GDNF were protective factors for preventing sleep disorders. Adenosine combined with GDNF had a higher diagnostic efficiency in predicting PD with sleep disorders by ROC analysis. This study revealed low adenosine and GDNF levels may be risk factors for sleep disorders in PD. The decrease of serum adenosine and GDNF levels may contribute to the diagnosis of PD with sleep disturbances.

## 1. Introduction

Parkinson’s disease (PD) is a neurological disease with a high associated morbidity, disability, and mortality. An increasing amount of research indicates PD is a multi-system disorder. The substantia nigra experiences an accumulation of α-synuclein and a loss of dopaminergic (DA) neurons as the typical pathological changes. Unilateral onset resting tremor, bradykinesia, hypertonia, abnormal posture, and gait are the characteristic motor symptoms. In addition, PD also has many non-motor symptoms such as constipation, cognitive disorders, sleep disturbances, and neuropsychiatric disorders, which increase the burden of disease. Among the non-motor symptoms, sleep disturbances are the most prevalent and affect 60–90% of patients. In some studies, insomnia, sleep fragmentation, and excessive daytime sleepiness (EDS) have been noted in over 50% of PD patients. Therefore, the early identification and comprehensive understanding of sleep disorders in PD can not only help screen people at high risk for PD but also provide better prognostic counseling, so that timely neuroprotective therapies can be initiated.

The pathogenesis of PD may be related to multiple factors, among which the neuroinflammatory response is key [1,2]. Abnormal activation of microglia in the central nervous system can contribute to the misfolding and aggregation of α-synuclein, resulting in neuronal damage. Activated microglia also secrete pro-inflammatory factors such as TNF-α, prostaglandin E2, interferon-γ, and interleukin-1β, which can adversely affect neuronal survival, and worsen mitochondrial dysfunction and oxidative stress responses. Mitochondrial dysfunction is the major pathological mechanism underlying the selective loss of nigrostriatal dopaminergic neurons in PD patients [3]. In addition, inflammation can lead to sleep disorders that then contribute to the abnormal expression of immune factors. During sleep-wake regulation, immune factors can act together with immunomodulators, namely adenosine (ADO), within the central nervous system [4].

Adenosine is a crucial neuromodulator in the central nervous system and is involved in many brain diseases such as PD. Adenosine can affect dopaminergic signal transduction in the brain by interacting with its receptors, regulating cognition, pain, sleep, and wakefulness, anxiety and depression, motor function, and other important physiological processes. Moreover, adenosine can control the release of DA, acetylcholine (Ach), serotonin (5-HT), and γ-aminobutyric acid (γ-GABA) by activating its receptors [5]. As an endogenous purine nucleoside, adenosine is a sleep-promoting substance. As shown in previous studies, adenosine or adenosine A1 receptor agonists administered intraventricularly or systemically in animals can significantly reduce wakefulness and increase sleep [6,7,8,9]. Conversely, caffeine and theophylline are both adenosine A1 and A_2A_ receptor antagonists and can reduce sleep [10,11]. Sleep time can be prolonged in rodents by inhibiting the activity of adenosine hydrolases such as adenosine kinase (ADK) and adenosine deaminase (ADA), reducing the breakdown of adenosine [12,13]. By inhibiting the activity of excitatory neurons, adenosine can mediate other neurotransmitters to regulate the sleep wakeful rhythm as follows: (a) Adenosine inhibits the neurotransmission of histaminergic neurons in the hypothalamic tuberomammillary nucleus to prolong the NREM sleep phase in rats [14]; (b) adenosine inhibits the action of cholinergic neurons in the brainstem and basal forebrain to promote sleep in rats [15]; (c) adenosine stimulates γ-GABA neurons in the ventrolateral preoptic region, promoting sleep in rats [16]; and (d) adenosine can reduce neuronal activity in the basal forebrain to promote the transition from awake to slow wave sleep [17,18]. Although it has been demonstrated that adenosine is crucial for normal sleep-wake regulation, its role in PD-associated sleep disorders has not been explored, and pathophysiological changes in PD adenosine levels and related neurotransmitters remain unclear.

By activating A_2B_ receptors, adenosine can also promote the transcription and release of glial cell line-derived neurotrophic factor (GDNF) in primary cultured rat astrocytes [19]. GDNF is a crucial player in enhancing DA neuron development, reducing apoptosis, and regulating cognitive function [20,21]. Basic research has shown that intracerebroventricular injection of GDNF can promote sleep by increasing the NREM sleep phase in rats and rabbits [22]. High-dose but not low-dose GDNF inhibits REM sleep in rabbits. However, elucidation of the role of GDNF in sleep is limited to basic research as no clinical studies have been conducted to test GDNF levels in PD patients with sleep disorders.

To address this limitation, we analyzed the associations of adenosine, GDNF, and related neurotransmitters and PD with sleep disorders. Furthermore, we examined the function of adenosine in PD with sleep disturbances, and its possible mechanisms, and attempted to identify potential indicators for the early prediction of PD with sleep disorders.

## 2. Materials and Methods

### 2.1. Participants

From June 2020 to March 2022, 87 patients with idiopathic PD (44 males and 43 females) were enrolled in the Department of Neurology and Outpatient at the Affiliated Hospital of Xuzhou Medical University. A total of 49 healthy individuals were recruited for the healthy control group from the Physical Examination Center of the same hospital; their age, nationality, gender, and educational years were matched with those in the PD group.

#### 2.1.1. Ethics Approval

The Medical Ethics Committee of Xuzhou Medical University’s Affiliated Hospital accepted this investigation (Approval No. XYFY2021-KL113-01). Everyone who took part signed an informed consent form.

#### 2.1.2. Inclusion and Exclusion Criteria

All PD individuals met the following criteria:

1. Aged ≥ 30 years;
2. Diagnosed with PD independently by two experienced neurologists using clinical diagnostic criteria from the UK Brain Bank [23] in combination with Movement Disorders Society (MDS) diagnostic criteria [24];
3. Normal listening, understanding and language expression, and could cooperate with clinicians in all neuropsychological, motor and mental behavior assessments;
4. Patient or a legal representative signed the informed consent form.

We excluded individuals with:

1. Other neurological diseases shown by imaging examination (CT or MRI), including various dementias, stroke, brain infection, tumor, or severe brain injury;
2. Secondary Parkinsonian syndrome (PS) or Parkinson-plus syndrome (P-PS) caused by trauma, infection, tumor, or drugs;
3. Severe systemic diseases (such as liver and kidney failure, circulatory system or blood system disorders);
4. Severe mental illness (such as schizophrenia, or bipolar disorder);
5. Other diseases that can cause sleep disorders (e.g., chronic poisoning, cancer pain, endocrine diseases, or urinary system diseases);
6. Incomplete data.

All healthy control individuals met the following criteria:

1. Understood the content and purpose of this study, willingly engaged in this study, and signed the informed consent form;
2. Able to complete all scale assessments under physician guidance;
3. Those with a Pittsburgh Sleep Quality Index (PSQI) ≤ 7 points.

We excluded individuals with:

1. Long-term alcoholism, smoking, drug abuse, or bad habits that seriously affect health (e.g., gambling addiction, unprotected sex, and sexually transmitted diseases);
2. A history of mental illness or sedative use;
3. Incomplete data.

### 2.2. Sample Collection and Testing

Venous blood samples were collected from all participants in vacuum blood collection tubes (Guangzhou Improve Medical Instruments Co., Ltd., Guangzhou, China) containing coagulant and separation gel. About 5 mL of fasting peripheral blood was drawn in the morning (fasting for at least 8 h). After standing at room temperature for up to 2 h, serum was separated by centrifugation (1000× *g*, 4 °C for 20 min), labeled, aliquoted, and stored at −80 °C. The levels of serum GDNF and 5-HT were quantified in the samples by enzyme-linked immunosorbent assay (ELISA) (CLOUD-CLONE Corp., Wuhan, China). The concentrations of serum ADO, GABA, and Ach were determined by liquid chromatography-mass spectrometry (LC-MS) (AB SCIEX, Framingham, MA, USA). Reference standards included ADO (CAS: 58-61-7) standard purity ≥ 99%, purchased from sigma Corporation, St. Louis, MO, USA; GABA (CAS: 56-12-2) standard purity ≥ 99%, purchased from Shanghai Aladdin Biochemical Technology Co., Ltd. Shanghai, China; and Ach (CAS: 60-31-1) standard purity ≥ 98%, purchased from Shanghai Macklin Biochemical Technology Co., Ltd., Shanghai, China. The concentrations of ADO, 5-HT, GABA, and Ach are expressed in ng/mL and the concentration of GDNF is expressed in pg/mL.

### 2.3. Medical Data Gathering

Two neurologists conducted face-to-face interviews to obtain demographic information such as gender, age, BMI, educational years, and medication. PD severity and motor symptoms were evaluated using the Hoehn-Yahr (H&Y) stage and the Movement Disorder Society-Unified Parkinson’s Disease Rating Scale III (MDS-UPDRS III) in the “ON” condition [25,26]. Standardized levodopa equivalent doses (LEDs) [27] were used to compare the medication status of PD patients. The non-motor Symptom Scale (NMSS) [28] was used to assess the frequency and degree of non-motor symptoms in various dimensions such as cardiovascular, cognitive, sleep, gastrointestinal and urinary. The Montreal Cognitive Assessment (MoCA) [29], 24-item Hamilton Depression Scale (HAMD) [30], 14-item Hamilton Anxiety Scale (HAMA) [31], REM sleep behavior disorder screening questionnaire (RBD-SQ) [32], Epworth Sleepiness Scale (ESS) [33], RLS diagnostic criteria [34], and Roman IV diagnostic criteria for constipation [35] were used to assess the patient’s cognition, depression, anxiety, REM sleep, daytime sleepiness, restless legs syndrome, and constipation, respectively. The HAMD and HAMA were assessed by the same psychologist.

In order to evaluate sleep quality, the Pittsburgh Sleep Quality Index (PSQI) [36] was adopted. This scale contains seven items, including subjective sleep quality, sleep latency, sleep duration, and so on. In Chinese population with sleep disorders, the sensitivity and specificity are the highest when the PSQI cutoff value is 7 points [37]. Therefore, the existence of a sleep disturbance was determined using a PSQI cut-off of > 7 points in this study. Based on the PSQI, the PD cohort was subgrouped into a PD with sleep disturbance group (PD-SD) and a PD without sleep disturbance group (PD-NSD). The PDSS scale [38] is used to evaluate EDS and issues with nocturnal sleep in PD patients. Scores range from 0 to 150 points; scores below 82 signify the existence of a sleep disturbance and are inversely associated with its severity.

### 2.4. Statistical Analysis

The Shapiro–Wilk test was used to determine whether the data were normal distribution. The indices that conformed to normal distribution were expressed as mean ± standard deviation ($\bar{x}\pm S$). Independent-samples *t*-test, one-way ANOVA, and LSD test were performed for comparisons between two groups, comparison among groups, and pairwise comparisons, respectively. Non-normally distributed data were expressed as median (quartile). Mann–Whitney U test, Kruskal–Wallis H test, and Nemenyi test were performed for comparisons between two groups, comparisons among groups, and pairwise comparisons, respectively. [*n* (%)] was used to express categorical count data. The Chi-squared test and the Chi-squared segmentation test were performed for comparisons between two groups, and pairwise comparisons, respectively. Each indicator and PSQI were compared using the Spearman correlation analysis. The correlation between influencing factors and sleep disorders in PD patients was examined using logistic regression. ROC curves were employed to analyze the diagnostic value of related indicators for sleep disorders. The statistical difference level was *p* < 0.05. GraphPad Prism v7 (San Diego, CA, USA) was used for generating plots and IBM SPSS v26.0 software (Armonk, NY, USA) for statistical analysis.

## 3. Results

### 3.1. Demographic Characteristics

A total of 49 patients in the HC group, 51 patients in the PD-SD group, and 36 patients in the PD-NSD group were included. The incidence of sleep disorders was 58.62%. Age, gender, education level, BMI, smoking, and drinking were found with no statistical discrepancies among groups (*p* > 0.05) (Table 1)

### 3.2. Non-Motor Symptoms and Sleep Disorders

Sleep disturbances are associated with many non-motor symptoms of PD, including autonomic nervous function, mood, and psychiatric symptoms. The results showed no substantial variations were observed in the average score of NMSS between the two PD groups (*p* = 0.11). To further explore whether other factors took part in influencing non-motor symptoms between groups, we further evaluated anxiety, depression, constipation, memory function, and other aspects.

#### 3.2.1. Cognitive and Sleep Disorders

**Figure 1:**
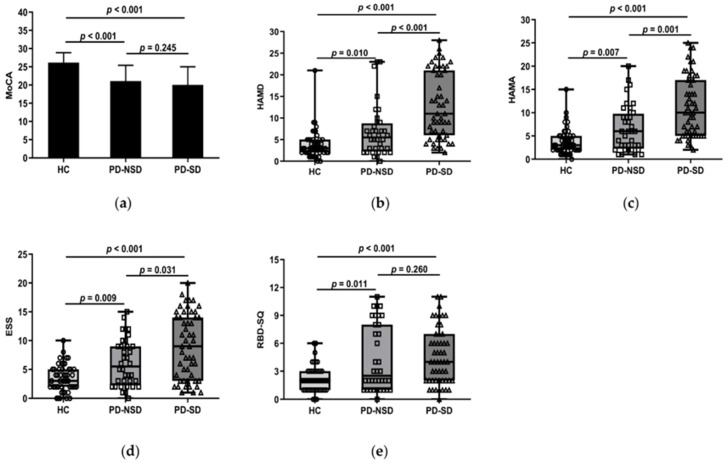
(**a**) Figure shows that the mean MoCA score of the HC group, 26.18 (SD 2.71), was higher than the PD-SD group, 20.04 (SD 4.97, *p* < 0.001), and the PD-NSD group, 21.08 (SD 4.32, *p* < 0.001), but no statistically relevant variations were found between PD-SD and PD-NSD groups (*p* = 0.245). (**b**) HAMD score in the PD-SD group (M 11, QR 6–21) was higher than in the PD-NSD group (M 5.50, QR 2.25–8.75, *p* < 0.001) and the HC group (M 3, QR 2–5, *p* < 0.001) respectively. Meanwhile, HAMD score in the PD-NSD group was higher than in the HC group (*p* = 0.01). (**c**) HAMA score (M 1, QR 5–17) in the PD-SD group was higher than in the PD-NSD group (M 6, QR 2.25–9.75, *p* = 0.001) and the HC group (M 3, QR 2–5, *p* < 0.001) respectively. HAMA score in the PD-NSD group was higher than in the HC group (*p* = 0.007). (**d**) ESS score in the PD-SD group (M 9, QR 3–14) was higher than in the PD-NSD group (M 5.5, QR 2.25–9, *p* = 0.031) and the HC group (M 3, QR 2–5, *p* < 0.001) respectively. Moreover, ESS score in the PD-NSD group was higher than in the HC group (*p* = 0.009). (**e**) RBD-SQ score (M 2, QR 1–3) of the HC group was lower than the PD-SD group (M 4, QR 2–7, *p* < 0.001) and the PD-NSD group (M 2.5, QR 1–8, *p* = 0.011) respectively, while there were no substantial discrepancies between PD groups (*p* = 0.260).

One-way ANOVA revealed the PD groups had lower scores of MoCA than the HC group (*p* < 0.05). But further LSD comparison showed there were no discrepancies between PD groups (*p* = 0.245) (Figure 1a).

#### 3.2.2. Anxiety, Depression, and Sleep Disorders

Kruskal–Wallis H test results found that the three groups’ HAMD and HAMA ratings varied (*p* < 0.05). Further comparison revealed the median HAMD of the PD-SD group was higher than that of the PD-NSD group (11 vs. 5.5, *p* < 0.001) and the HC group (11 vs. 3, *p* < 0.001) (Figure 1b). The median HAMA in the PD-SD group (10 points) was higher than the PD-NSD group (6 points, *p* = 0.001) and the HC group (3 points, *p* < 0.001) (Figure 1c). This indicates anxiety and depression levels of PD-SD patients were more serious than those without sleep disorders and normal controls.

#### 3.2.3. Constipation and Sleep Disorders

The Chi-Squared test showed the proportion of constipation in the PD-SD group (60.8%) and the PD-NSD group (58.3%) was considerably higher than in the HC group (28.6%) (*p* < 0.05). However, no variations were found between PD groups.

#### 3.2.4. Comparison of PDSS, ESS, RBD-SQ, and RLS among Groups

In this study, the median PDSS in the PD-SD group was 79, which was lower than 129 in the PD-NSD group (*p* < 0.001). Lower scores in the PDSS indicate more severe sleep disturbances. The average ESS score of the PD-SD group was 9, which was higher than the PD-NSD group (5.5, *p* = 0.031) and the HC group (3, *p* < 0.001) (Figure 1d). Moreover, pairwise comparisons of the three groups showed significant differences. It implied that EDS in the PD-SD group is more severe than in the other two groups. The total score of RBD-SQ was not consistent in each group. The PD-SD and PD-NSD group had higher RBD-SQ scores than the HC group (*p* < 0.05), but no discrepancies were observed between PD groups (*p* = 0.26) (Figure 1e). About 13 patients (25.5%) in the PD-SD group presented with RLS, compared to 3 patients (6.1%) in the HC group (*p* = 0.031). There were 6 cases (16.7%) in the PD-NSD group combined with RLS, with no significant discrepancies discovered between the PD-NSD group and the other two groups (*p* > 0.05).

### 3.3. Motor Symptoms and Sleep Disorders

Independent sample *t*-test showed the PD-SD group had higher mean UPDRS-III scores (30.98) than the PD-NSD group (22.67) (*p* = 0.013). H-Y stage in the PD-SD group (median stage 2.5) was higher than in PD-NSD group (median stage 2), according to the Mann–Whitney U test (*p* < 0.001). The findings suggested more severe motor symptoms may appear when sleep disturbances start. At the same time, we looked into how sleep disturbances would influence disease duration. Our results found the course of disease in the PD-SD group (median 66 months) was longer than in the PD-NSD group (median 54 months), but there were no variations between the two groups (*p* = 0.146). Likewise, no discernible discrepancies were observed in the number of tremor-predominant, tonic-predominant, and mixed types between PD groups (*p* > 0.05).

### 3.4. Antiparkinsonian Medications between PD Groups

There was no discernible difference in LED dosage between PD-SD and PD-NSD groups, according to the Mann–Whitney U test (*p* > 0.05). The Chi-Squared test also showed no substantial difference in levodopa, dopamine agonist, MAO-B inhibitors, and amantadine hydrochloride between the two groups (Table 2).

### 3.5. Levels of Serum Adenosine, GDNF, and Related Neurotransmitters in Each Group

**Figure 2:**
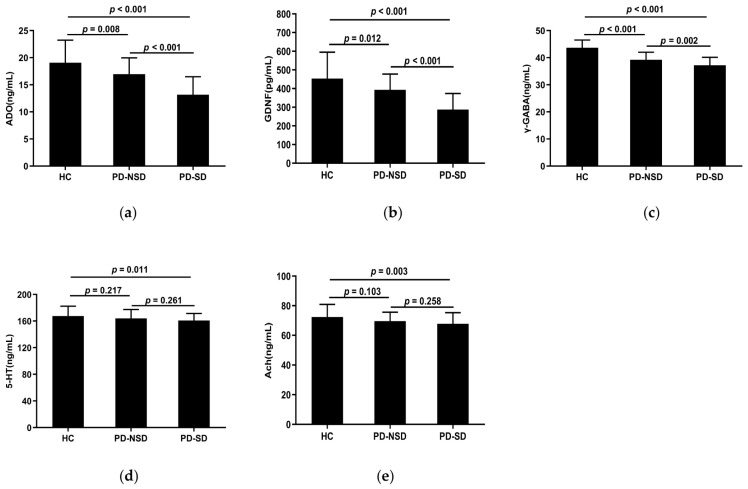
(**a**) The mean ADO levels of the PD-SD group (M 13.18, SD 3.31) were lower than the PD-NSD group (M 16.96, SD 3.01, *p* < 0.001) and the HC group (M 19.07, SD 4.16, *p* < 0.001) respectively, with statistical difference between the PD-NSD group and the HC group (*p* = 0.008). (**b**) The levels of GDNF in the PD-SD group (M 287.55, SD 85.92) were lower than in the PD-NSD group (M 392.81, SD 85.08, *p* < 0.001) and the HC group (M 453.56, SD 141.26, *p* < 0.001), respectively. Meanwhile, the PD-NSD group had lower GDNF levels than the HC group (*p* = 0.012). (**c**) The levels of γ-GABA in the PD-SD group (M 37.21, SD 2.94) were lower than in the PD-NSD group (M 39.22, SD 2.79, *p* = 0.002) and the HC group (M 43.68, SD 2.84, *p* < 0.001) respectively, with variations between the PD-NSD group and the HC group (*p* < 0.001). (**d**) The PD-SD group had lower mean 5-HT levels (M 160.9, SD 10.52) than the PD-NSD group (M 164.08, SD 13.33, *p* = 0.261) and the HC group (M 167.61, SD 14.88, *p* = 0.011), while there was no statistical difference between PD groups (*p* = 0.261). (**e**) Ach levels in the PD-SD group (M 67.78, SD 7.50) were lower than in the PD-NSD group (M 69.64, SD 5.99, *p* = 0.258) and the HC group (M 72.36, SD 8.53, *p* = 0.003) respectively, but no substantial difference between PD-SD and PD-NSD groups (*p* = 0.258).

Table 3 displays the levels of serum detection indices in each group. The serum concentrations of ADO, GDNF, 5-HT, γ-GABA, and Ach in the three groups were substantially different according to one-way ANOVA (*p* < 0.05). The LSD pairwise comparison revealed ADO, GDNF, and γ-GABA levels in the PD-SD group (13.18 ng/mL, 287.55 pg/mL, 37.21 ng/mL) were lower than those in the PD-NSD group (16.96 ng/mL, 392.81 pg/mL, 39.22 ng/mL) and HC group (19.07 ng/mL, 453.56 pg/mL, 43.68 ng/mL) respectively (Figure 2a–c). Additionally, pairwise comparisons among the three groups showed statistical significance (*p* < 0.05). The levels of 5-HT and Ach in the PD-SD group (160.9 ng/mL, 67.78 ng/mL) were lower than in the HC group (167.61 ng/mL, 72.36 ng/mL) (*p* < 0.05). However, there were no variations between the PD-NSD and HC groups, nor the PD-SD and PD-NSD groups (Figure 2d,e).

### 3.6. Correlation Analysis of Each Index and the PSQI Scale in PD Patients

#### 3.6.1. The Correlations between ADO, GDNF, γ-GABA, 5-HT, Ach, and the PSQI Scale

**Figure 3:**
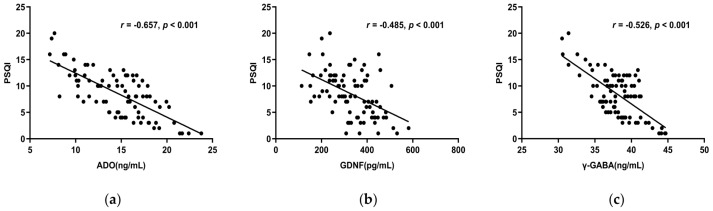
(**a**) The total score of PSQI and serum ADO concentration had a negative correlation (*r* = −0.657, *p* < 0.001). (**b**) The PSQI overall score and serum GDNF levels had a negative connection (*r* = −0.485, *p* < 0.001). (**c**) The PSQI total score and serum γ-GABA levels revealed a negative connection (*r* = −0.526, *p* < 0.001). Abbreviations: PSQI: Pittsburgh Sleep Quality Index.

Spearman correlation analysis showed ADO, GDNF, and γ-GABA, all correlated negatively with the PSQI total score (*r* < 0, *p* < 0.05) (Figure 3a–c), whereas 5-HT and Ach did not (*p* > 0.05). The PSQI scale’s subjective sleep quality, sleep latency, sleep duration, habitual sleep efficiency, and use of sleeping medication were all substantially adversely linked with ADO, GDNF, and γ-GABA. Furthermore, ADO and γ-GABA had a negative correlation with daytime dysfunction as well (Table 4). This suggests lower serum concentrations of ADO, GDNF, and γ-GABA indicate decreased sleep quality.

#### 3.6.2. The Correlations between MoCA, HAMA, HAMD, UPDRS-III, H-Y, Course of the Disease, LED, RBD-SQ, and the PSQI Scale

**Figure 4:**
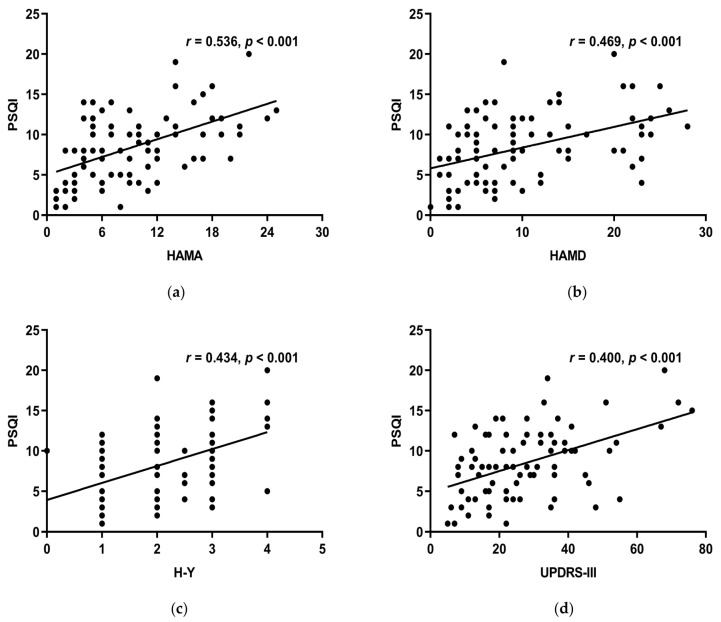
(**a**) HAMA and the PSQI overall score had a positive connection (*r* = 0.536, *p* < 0.001). (**b**) HAMD and the total score of PSQI had a positive connection (*r* = 0.469, *p* < 0.001). (**c**) H-Y stage and the PSQI total score had a positive connection (*r* = 0.434, *p* < 0.001). (**d**) Showed a positive connection between UPDRS-III and PSQI total score (*r* = 0.400, *p* < 0.001).

HAMA, HAMD, H-Y stage, and UPDRS-III all linked positively with the PSQI total score (*r* > 0, *p* < 0.05) (Figure 4a–d), while MoCA, course of the disease, LED, RBD-SQ did not (*p* > 0.05). HAMA, HAMD, and UPDRS-III had a positive correlation with subjective sleep quality, sleep latency, sleep duration, habitual sleep efficiency, use of sleeping medication, and daytime dysfunction of the PSQI scale. Moreover, the H-Y stage had a positive correlation with all the other components except for sleep latency (Table 5). This suggests that decreased sleep quality indicates more noticeable motor symptoms, more serious conditions, more severe anxiety, and depression.

### 3.7. Risk Factors of Sleep Disturbances

Binary logistic regression was set up with the presence of sleep disorders in PD patients as the dependent variable (yes = 1, no = 0), and GDNF, ADO, GABA, HAMD, HAMA, UPDRS-III, and H-Y stage as the independent variable. The results showed GDNF, ADO, and HAMD had statistical difference in the model (*p* < 0.05) (Table 6). Given this, depression was a risk factor for PD patients with sleep disorders (OR > 1, *p* < 0.05), and GDNF and ADO were protective factors in preventing sleep disorders (OR < 1, *p* < 0.05).

### 3.8. The ROC Curve Analysis of Serum ADO and GDNF

**Figure 5:**
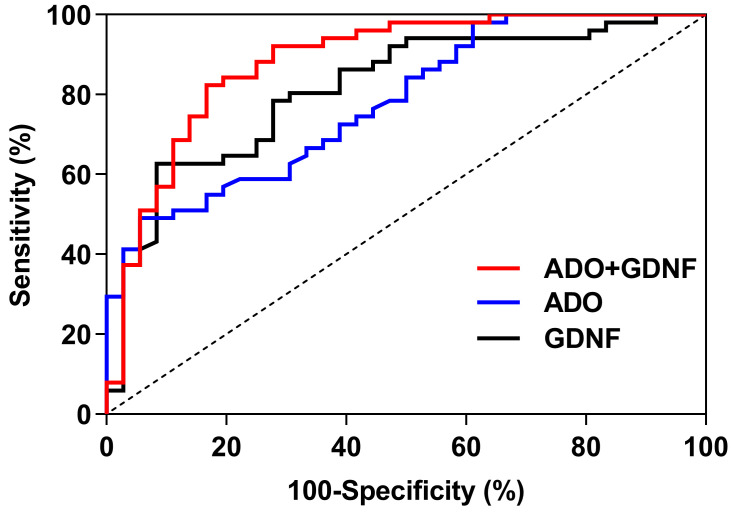
ROC curves of ADO and GDNF.

For predicting sleep disorders, the GDNF, ADO, and ADO + GDNF areas under the curve were 0.815, 0.780, and 0.883 respectively (*p* < 0.001) (Figure 5). The threshold of GDNF in predicting sleep disorders was 305.205, with a sensitivity of 62.7% and a specificity of 91.7%. With a sensitivity of 49.0% and a specificity of 94.4%, the ADO threshold for predicting sleep disorders was 13.000. ADO + GDNF had a threshold of 0.570 for detecting sleep problems, with equivalent sensitivity and specificity of 82.4% and 83.3% (Table 7).

## 4. Discussion

The characteristic pathological features of PD mainly include misfolded amyloid inclusions in the substantia nigra pars compacta, i.e., Lewy bodies, where the major component is α-synuclein. Horsager et al. [39] found that α-synuclein initially appears in the intestinal or peripheral autonomic nervous system in body-first PD; the vagus nerve, which innervates internal organs such as the gastrointestinal tract, helps these toxic proteins to be transported to the brain through nerve branches. Therefore, non-motor symptoms including hyposmia, constipation, sleep disturbance, emotional disorder, and cognitive disorder in Braak stages 1–2 [40] (i.e., the prodromal stage that arises 5–20 years before the clinical onset of PD) have occurred before the damage to the substantia nigra of the brain is evident. Among these disease manifestations, sleep problems are the most prevalent non-motor symptom in the preclinical stage of PD; patients with PD experience sleep disorders at a much higher incidence than healthy age-matched individuals. The incidence rate of PD with sleep disorders was 58.62% in this study; that is, over half of our PD patients suffered from a sleep disorder, seriously affecting their quality of life.

As reported in previous studies, α-synuclein is present in neurons and astrocytes [41]. As one of the most widely distributed cells in the brain, astrocytes act as a key function in regulating cerebral vascular tension, modulating the blood–brain barrier’s permeability, regulating neuronal activity and synaptic function, maintaining sleep homeostasis, and regulating the dynamic balance of neurotransmitters. Under normal circumstances, astrocytes are in a resting state. In neurodegenerative diseases such as PD, astrocytes transition to an activated state, and their numbers increase. At the beginning of the illness, activated astrocytes can secrete a large number of neurotrophic factors, which have a protective effect on DA neurons. As the disease progresses, astrocytes become cytotoxic, worsening oxidative stress and inflammation, reducing GDNF levels, and inducing apoptosis through endoplasmic reticulum stress [42]. In PD, α-synuclein aggregated in astrocytes likely originates from nerve cells and has undergone protein misfolding and other changes before being released from the axon terminals of affected neurons contributing to the degeneration of DA neurons [43,44].

The modulation of DA and other neurotransmitters in the brain is mediated by adenosine and its receptors, but adenosine’s involvement in PD with sleep disorders has not been reported. The levels of serum adenosine and γ-GABA in the PD-SD group were lower than those in the PD-NSD and HC groups, according to our work. However, the levels of 5-HT and Ach were not different across the PD groups. Adenosine and γ-GABA were adversely linked with the PSQI total score and all of its components except subitem 5. These findings suggest that lower serum adenosine and γ-GABA correspond to worse quality of sleep. As the main source of ATP and adenosine, astrocytes can rapidly convert ATP into adenosine by active and passive release and concentration-dependent clearance, regulating the level of extracellular adenosine [45,46,47]. Adenosine can also act on the sleep regulation center or cerebral cortex by binding to activated receptors, thereby enhancing slow-wave activity and maintaining sleep homeostasis [48]. As PD develops, however, a large number of DA neurons are degenerated and necrotic, there is a high expression of α-synuclein, and the normal function of astrocytes is disrupted, resulting in a further decrease in adenosine production. At the same time, the striatum is rich in adenosine A_2A_ receptors, so with the decrease in striatal DA in PD, the regulation of adenosine and its receptors on other neurotransmitters and sleep is affected, thus breaking the homeostatic regulation of sleep.

As an inhibitory neurotransmitter, γ-GABA is produced by the decarboxylation of glutamate (Glu) by decarboxylase, which may be responsible for regulating wakefulness and sleep. Serum γ-GABA levels were lower in the PD-SD group and negatively correlated with sleep quality in this study, consistent with previous reports [49]. Glu and GABA are responsible for regulating the conversion of NREM and REM during sleep. Animal research has demonstrated the GABAergic (γ-GABA release) pathway in the ventral medulla can contribute to REM sleep in mice [50]. Watson et al. [51] found that decreased GABA levels in the pontine reticular formation lead to decreased REM sleep and increased wakefulness in rats. In addition, adenosine regulation of sleep varies from brain region to brain region. In the ventrolateral preoptic area of the hypothalamus, adenosine can activate GABA neurons to induce and maintain sleep [12]. By increasing the release of GABA in the tuberomammillary nucleus, adenosine can inhibit histaminergic neurons to promote sleep [52]. In PD, however, mitochondrial damage leads to not only a decrease of adenosine synthesis but also a loss of neurons in the substantia nigra pars compacta by increasing Ca^2+^ excitotoxicity; the function of γ-GABA, which regulates Ca^2+^ influx, is also impaired, further worsening sleep disorders [53].

Using logistic regression analysis, we found that GDNF and adenosine were protective factors in PD patients with sleep disorders. Lower concentrations of GDNF and adenosine were associated with more serious sleep disorders. Decreased GDNF levels are related to the pathophysiological mechanisms of PD. In PD, activated astrocytes can produce GDNF, but the Ca^2+^/GABA system breaks down in the presence of toxic effects such as oxidative stress and calcium overload, worsening the death of DA neurons, and GDNF regulated by the Ca^2+^/GABA system is further reduced. At the same time, GDNF can not only promote the differentiation and survival of DA neurons, but also regulate the production and release of neurotransmitters by DA neurons and serotonergic neurons [54]. It is widely accepted that DA and 5-HT are involved in controlling sleep and promoting wakefulness, and that 5-HT can increase sleep in some cases, but that these transmission systems are impaired in PD [55]. Studies have shown that sleep duration and sleep architecture are changed in MPTP-treated mice with PD, as manifested by decreased wakefulness and increased REM sleep [56]. Decker et al. [57] found that the destruction of DA neurons in the ventral tegmental area, substantia nigra, and periaqueductal gray matter can also result in similar changes in rats. Loss of dopaminergic cells can impair the wakefulness mechanism by other means than the nigrostriatal system [58], and lots of PD patients have a short REM sleep latency during daytime naps. 5-HT takes part in regulating wakefulness, slow-wave sleep, and REM sleep. Research on animals have revealed that raphe nucleus injury leads to decreased sleep, which is related to decreased 5-HT levels [59]. Hipólide et al. discovered after 96 h of sleep deprivation, 5-HT binding decreased in certain brain regions, and its receptor was down-regulated; this may be related to increased synaptic 5-HT [60]. Decreased GDNF in PD patients with sleep disorders can affect the regulation of these neurotransmitters, thereby worsening sleep disorders.

In addition, it was found previously that orexin, an excitatory neurotransmitter that regulates wakefulness, is associated with PD with sleep disorders, especially excessive daytime sleepiness [61]. The results of analyses of postmortem brain tissue from PD patients and cerebrospinal fluid examinations of living PD patients suggest a decrease in orexin and a negative correlation with disease severity [61,62,63,64]. The two possible reasons are as follows: (1) Orexin is a polypeptide secreted by dorsal and lateral hypothalamic neurons, and it has been reported in some studies that the α-synuclein load in the hypothalamic area significantly increases in PD patients with sleep disorders [65], and that disruption of this area may lead to a decrease in orexin; (2) the activity of orexin neurons is regulated by DA [66] and other neurotransmitters (e.g., γ-GABA [67], and serotonin [68]), and disruption of these neurotransmitter systems in PD with sleep disorders leads to disturbances in orexin levels. However, a retrospective study published by Ogawa et al. [69] in 2022 showed that decreased orexin in cerebrospinal fluid is not associated with PD with sleep disorders; this finding may have been biased by patient medication, the short follow-up, and various complex pathophysiological mechanisms of PD.

Some investigators have reported that dopaminergic drugs can increase the risk of PD with sleep disorders and that an increase in the DA dose is significantly associated with decreased sleep quality [70]. In addition, some drugs (such as selegiline) may increase the risk of insomnia and reduce sleep continuity and REM sleep. Levodopa and DA agonists (e.g., pramipexole, ropinirole, and rotigotine) cause somnolence [71,72]. However, in this study, we discovered no discernible changes in the number of patients using LEDs, levodopa, DA agonists, MAO-B inhibitors, and amantadine between the PD-SD and PD-NSD groups. The French CoPark cohort conducted a cross-sectional study in which LEDs were found to not be associated with sleep disorders [73]. The reason for these contradictory results may be related to the different severities of PD between their study and ours, and the different assessment methods utilized to assess sleep disorders (subjective vs. objective). Therefore, the types of sleep disorders and drug dosage should be further tested in the future, and a larger sample size and a longer follow-up period are needed to confidently ascertain generalizable relationships.

We also found that more severe anxiety, depression, motor dysfunction, and PD condition were related to poorer sleep quality. Depression is a risk factor in PD patients with sleep disorders. Nocturnal motor symptoms and psychiatric symptoms in PD patients can interfere with the sleep architecture and produce motor fluctuations, thereby affecting sleep quality. Studies have reported that shortened sleep time in PD patients is closely related to depressive symptoms, which may be associated with DA dysfunction [74]. At the same time, as PD progresses, the decrease in neurotransmitters such as DA and 5-HT further aggravates motor dysfunction and mental symptoms, leading to sleep interruption.

## 5. Conclusions

In conclusion, we performed a multidimensional assessment of PD with sleep disorders; our findings provide a basis for the early detection and understanding of sleep disturbances. At present, the diagnosis of PD with sleep disturbances depends on polysomnography (PSG), but PSG is difficult to implement in clinical practice due to patient discomfort and iatrogenic effects on sleep quality during the test. We found low levels of adenosine and GDNF were risk factors for PD with sleep disorders in this study. Adenosine combined with GDNF showed high diagnostic efficacy for PD with sleep disorders. Studying the interactions between adenosine, GDNF, and related neurotransmitters may provide better therapeutic strategies for sleep disturbances and a novel understanding of the pathogenesis of PD with sleep disorders. It is important to note some limitations of this study. First, the changes in each indicator and sleep disorders over time could not be determined in this cross-sectional study, so future research should apply more dynamic follow-up observations to verify our findings. Second, the sample size was relatively small, so larger-sample and multicenter studies are needed in the future to test the generalizability of our findings. Third, considering the heterogeneity of PD patients, deeper studies are needed to rule out the impact of other factors. Moreover, the relationships between different types of sleep disorders and neurotransmitters need to be further characterized and imaging examinations such as high-resolution MRI should be carried out to define the best direction for the early diagnosis of PD with sleep disorders.

## Figures and Tables

**Table 1:** Demographic characteristics and clinical data in each group.

Information	HC (*n* = 49)	PD-NSD (*n* = 36)	PD-SD (*n* = 51)	Statistic	*p*
Age (years)	65.57 ± 8.85	66.86 ± 8.9	67.57 ± 8.75	0.652 ^a^	0.523
Gender (%)				0.279 ^b^	0.870
Male	25 (51.0)	17 (47.2)	27 (52.9)		
Female	24 (49.0)	19 (52.8)	24 (47.1)		
Education (years)	7.00 (6.00, 9.00)	7.50 (6.00, 9.00)	7.00 (6.00, 9.00)	0.029 ^c^	0.985
BMI (kg/m^2)^	24.96 ± 3.19	24.07 ± 2.76	24.21 ± 3.16	1.119 ^a^	0.330
Smoking history (%)	6 (12.2)	6 (16.7)	10 (19.6)	1.008 ^b^	0.604
Alcohol consumption (%)	9 (18.4)	7 (19.4)	10 (19.6)	0.028 ^b^	0.986
MoCA (scores)	26.18 ± 2.71	21.08 ± 4.32 *	20.04 ± 4.97 *	31.021 ^a^	<0.001
HAMD (scores)	3.00 (2.00, 5.00)	5.50 (2.25, 8.75) *	11.00 (6.00, 21.00) *^,#^	47.003 ^c^	<0.001
HAMA (scores)	3.00 (2.00, 5.00)	6.00 (2.25, 9.75) *	10.00 (5.00, 17.00) *^,#^	45.217 ^c^	<0.001
RBD-SQ (scores)	2.00 (1.00, 3.00)	2.50 (1.00, 8.00) *	4.00 (2.00, 7.00) *	16.746 ^c^	<0.001
ESS (scores)	3.00 (2.00, 5.00)	5.50 (2.25, 9.00) *	9.00 (3.00, 14.00) *^,#^	27.448 ^c^	<0.001
RLS, case (%)	3 (6.1)	6 (16.7)	13 (25.5) *	6.922 ^b^	0.031
Constipation, case (%)	14 (28.6)	21 (58.3) *	31(60.8) *	12.266 ^b^	0.002
UPDRS-III (scores)	NA	22.67 ± 12.97	30.98 ± 16.26	2.547 ^d^	0.013
NMSS (scores)	NA	21.00 (15.25, 64.00)	41.00 (20.00, 74.00)	−1.599 ^e^	0.110
Phenotype (%)				0.746 ^b^	0.689
TD	NA	20 (55.6)	24 (47.1)		
PIGD	NA	11 (30.6)	20 (39.2)		
Indeterminate	NA	5 (13.9)	7 (13.7)		
Disease duration (months)	NA	54.00 (26.75, 72.00)	66.00 (48.00, 82.00)	−1.452 ^e^	0.146
PDSS (scores)	NA	129.00 (102.00, 139.00)	79.00 (72.00, 81.00)	−7.110 ^e^	<0.001
H-Y (on-stage)	NA	2.00 (1.00, 2.50)	2.50 (2.00, 3.00)	−2.716 ^e^	<0.001

Note: NA, not available. The * symbol denotes compared with the HC group, *p* < 0.05. The ^#^ symbol denotes compared with the PD-NSD group, *p* < 0.05. One-way ANOVA was used to analyze data in ^a^, the Chi-square test was used as ^b^, the Kruskal–Wallis H test was used as ^c^, the independent sample *t*-test was used as ^d^, and the Mann–Whitney U test was used as ^e^. Abbreviations: HC: healthy control group; PD-SD: Parkinson’s disease with sleep disorders group; PD-NSD: Parkinson’s disease without sleep disorders group; MoCA: Montreal cognitive assessment; HAMD: Hamilton depression scale; HAMA: Hamilton anxiety scale; RBD-SQ: REM sleep behavior disorder screening questionnaire; ESS: Epworth sleepiness scale; RLS: Restless legs syndrome; UPDRS-III: Movement disorder society-unified Parkinson’s disease rating scale III; NMSS: non-motor symptom scale; PDSS: Parkinson’s disease sleep scale; H-Y: Hoehn-Yahr stage.

**Table 2:** Antiparkinsonian medications between PD groups.

Drug	PD-NSD (*n* = 36)	PD-SD (*n* = 51)	*Z*/*χ*^2^	*p*
LED (mg)	412.50 (337.50, 596.88)	425.00 (337.50, 575.00)	−0.108	0.914
Levodopa/benserazide (%)	32 (88.9)	48 (94.1)	0.233	0.629
Dopamine agonist (%)	24 (66.7)	38 (74.5)	0.634	0.426
MAO-B inhibitors (%)	12 (33.3)	20 (39.2)	0.314	0.575
Amantadine hydrochloride (%)	4 (11.1)	12 (23.5)	2.168	0.141

Note: Abbreviations: LED: levodopa equivalent dose; MAO-B: mono amine oxydase.

**Table 3:** The levels of serum detection indexes among groups.

Test Indexes	HC (*n* = 49)	PD-NSD (*n* = 36)	PD-SD (*n* = 51)	*F*	*p*
ADO (ng/mL)	19.07 ± 4.16	16.96 ± 3.01 *	13.18 ± 3.31 *^,#^	34.792	<0.001
GDNF (pg/mL)	453.56 ± 141.26	392.81 ± 85.08 *	287.55 ± 85.92 *^,#^	29.619	<0.001
5-HT (ng/mL)	167.61 ± 14.88	164.08 ± 13.33	160.9 ± 10.52 *	3.351	0.038
γ-GABA (ng/mL)	43.68 ± 2.84	39.22 ± 2.79 *	37.21 ± 2.94 *^,#^	66.045	<0.001
Ach (ng/mL)	72.36 ± 8.53	69.64 ± 5.99	67.78 ± 7.50 *	4.657	0.011

Note: The * symbol denotes compared with the HC group, *p* < 0.05. The ^#^ symbol denotes compared with the PD-NSD group, *p* < 0.05. Abbreviations: ADO: adenosine; GDNF: glial cell line-derived neurotrophic factor; 5-HT: serotonin; γ-GABA: γ-aminobutyric acid; Ach: acetylcholine.

**Table 4:** Correlation between ADO, GDNF, γ-GABA, 5-HT, Ach, and PSQI scale.

Indicators		PSQI Total Score	Component 1	Component 2	Component 3	Component 4	Component 5	Component 6	Component 7
GDNF	*r*	−0.485 **	−0.375 **	−0.340 **	−0.381 **	−0.428 **	−0.164	−0.315 **	−0.175
	*p*	<0.001	<0.001	0.001	<0.001	<0.001	0.128	0.003	0.105
5-HT	*r*	−0.029	0.110	0.074	−0.154	−0.078	−0.053	0.114	−0.039
	*p*	0.793	0.308	0.497	0.154	0.470	0.625	0.292	0.720
ADO	*r*	−0.657 **	−0.479 **	−0.492 **	−0.494 **	−0.438 **	−0.108	−0.502 **	−0.484 **
	*p*	<0.001	<0.001	<0.001	<0.001	<0.001	0.318	<0.001	<0.001
γ-GABA	*r*	−0.526 **	−0.510 **	−0.351 **	−0.325 **	−0.277 **	−0.131	−0.468 **	−0.583 **
	*p*	<0.001	<0.001	0.001	0.002	0.009	0.226	<0.001	<0.001
Ach	*r*	−0.191	−0.280 **	−0.138	−0.053	−0.050	−0.209	−0.123	−0.149
	*p*	0.076	0.009	0.204	0.628	0.643	0.052	0.256	0.169

Note: The ** symbol denotes *p* < 0.01. Component 1 is subjective sleep quality. Component 2 is sleep latency. Component 3 is sleep duration. Component 4 is habitual sleep efficiency. Component 5 is sleep disturbances. Component 6 is the use of sleeping medication. Component 7 is daytime dysfunction.

**Table 5:** Correlation between each clinical index and PSQI scale.

Indicators		PSQI Total Score	Component 1	Component 2	Component 3	Component 4	Component 5	Component 6	Component 7
MoCA	*r*	−0.164	−0.216 *	0.050	−0.095	−0.127	−0.242 *	−0.045	−0.229 *
	*p*	0.130	0.044	0.644	0.380	0.240	0.024	0.679	0.033
HAMA	*r*	0.536 **	0.418 **	0.264 *	0.460 **	0.356 **	0.168	0.336 **	0.468 **
	*p*	<0.001	<0.001	0.014	<0.001	0.001	0.121	0.001	<0.001
HAMD	*r*	0.469 **	0.396 **	0.289 **	0.370**	0.335 **	0.193	0.269 *	0.255 *
	*p*	<0.001	<0.001	0.007	<0.001	0.002	0.073	0.012	0.017
UPDRS-III	*r*	0.400 **	0.334 **	0.432 **	0.246 *	0.270 *	0.027	0.280 **	0.231 *
	*p*	<0.001	0.002	<0.001	0.022	0.011	0.802	0.009	0.031
H-Y stage	*r*	0.434 **	0.316 **	0.115	0.261 *	0.354 **	0.326 **	0.248 *	0.452 **
	*p*	<0.001	0.003	0.288	0.015	0.001	0.002	0.020	<0.001
The course of disease	*r*	0.064	−0.003	0.014	0.083	0.108	−0.137	0.084	0.014
	*p*	0.557	0.976	0.899	0.442	0.321	0.207	0.439	0.894
LED	*r*	0.205	0.273 *	0.059	0.108	0.044	0.101	0.202	0.322 **
	*p*	0.056	0.011	0.590	0.319	0.684	0.351	0.061	0.002
RBD-SQ	*r*	0.178	0.070	−0.100	0.060	0.208	0.224 *	0.099	0.339 **
	*p*	0.100	0.521	0.359	0.581	0.053	0.037	0.361	0.001

Note: The * symbol denotes *p* < 0.05. The ** symbol denotes *p* < 0.01.

**Table 6:** Logistic regression of sleep disorders in PD patients.

Factors	*B*	*SE*	*Waldχ* ^2^	*p*	*OR* (95% *CI*)
GDNF	−0.017	0.005	12.404	<0.001	0.983 (0.974~0.993)
ADO	−0.577	0.211	7.464	0.006	0.562 (0.371~0.85)
γ-GABA	0.441	0.246	3.202	0.074	1.554 (0.959~2.519)
HAMD	0.128	0.059	4.709	0.030	1.137 (1.013~1.277)
HAMA	0.001	0.072	0.001	0.984	1.001 (0.87~1.153)
UPDRS-III	0.001	0.025	0.002	0.963	1.001 (0.953~1.052)
H-Y stage	−0.096	0.393	0.059	0.807	0.909 (0.421~1.962)
Constant	−3.211	7.317	0.193	0.661	-

**Table 7:** ROC analysis of ADO and GDNF.

Parameter	GDNF	ADO	ADO + GDNF
*AUC* 95% *CI*	0.815 0.723~0.906	0.780 0.685~0.875	0.883 0.808~0.959
*SE*	0.047	0.048	0.039
*p*	<0.001	<0.001	<0.001
The threshold value	305.205	13.000	0.570
Sensitivity (%)	62.7	49.0	82.4
Specificity (%)	91.7	94.4	83.3

## Data Availability

The data supporting the findings of this study are included in the article, further inquiries can be directed to the corresponding authors.
